# Integrated lncRNA and mRNA Transcriptome Analyses in the Ovary of *Cynoglossus semilaevis* Reveal Genes and Pathways Potentially Involved in Reproduction

**DOI:** 10.3389/fgene.2021.671729

**Published:** 2021-05-19

**Authors:** Yani Dong, Likang Lyu, Daiqiang Zhang, Jing Li, Haishen Wen, Bao Shi

**Affiliations:** ^1^Key Laboratory of Mariculture, Ministry of Education, Fisheries College, Ocean University of China, Qingdao, China; ^2^Key Laboratory of Sustainable Development of Marine Fisheries, Ministry of Agriculture and Rural Affairs, Yellow Sea Fisheries Research Institute, Chinese Academy of Fishery Sciences, Qingdao, China; ^3^Laboratory for Marine Fisheries and Food Production Processes, Pilot National Laboratory for Marine Science and Technology, Qingdao, China

**Keywords:** *Cynoglossus semilaevis*, dual-fluorescence *in situ* hybridization, transcriptome, integrated analysis, reproduction, ovary, mRNA, lncRNA

## Abstract

Long non-coding RNAs (lncRNAs) have been reported to be involved in multiple biological processes. However, the roles of lncRNAs in the reproduction of half-smooth tongue sole (*Cynoglossus semilaevis*) are unclear, especially in the molecular regulatory mechanism driving ovarian development and ovulation. Thus, to explore the mRNA and lncRNA mechanisms regulating reproduction, we collected tongue sole ovaries in three stages for RNA sequencing. In stage IV vs. V, we identified 312 differentially expressed (DE) mRNAs and 58 DE lncRNAs. In stage V vs. VI, we identified 1,059 DE mRNAs and 187 DE lncRNAs. Gene Ontology (GO) and Kyoto Encyclopedia of Genes and Genomes (KEGG) enrichment analyses showed that DE mRNAs were enriched in ECM-receptor interaction, oocyte meiosis and steroid hormone biosynthesis pathways. Furthermore, we carried out gene set enrichment analysis (GSEA) to identify potential reproduction related-pathways additionally, such as fatty metabolism and retinol metabolism. Based on enrichment analysis, DE mRNAs with a potential role in reproduction were selected and classified into six categories, including signal transduction, cell growth and death, immune response, metabolism, transport and catabolism, and cell junction. The interactions of DE lncRNAs and mRNAs were predicted according to *antisense*, *cis*-, and *trans-*regulatory mechanisms. We constructed a competing endogenous RNA (ceRNA) network. Several lncRNAs were predicted to regulate genes related to reproduction including *cyp17a1*, *cyp19a1, mmp14, pgr*, and *hsd17b1.* The functional enrichment analysis of these target genes of lncRNAs revealed that they were involved in several signaling pathways, such as the TGF-beta, Wnt signaling, and MAPK signaling pathways and reproduction related-pathways such as the progesterone-mediated oocyte maturation, oocyte meiosis, and GnRH signaling pathway. RT-qPCR analysis showed that two lncRNAs (XR_522278.2 and XR_522171.2) were mainly expressed in the ovary. Dual-fluorescence *in situ* hybridization experiments showed that both XR_522278.2 and XR_522171.2 colocalized with their target genes *cyp17a1* and *cyp19a1*, respectively, in the follicular cell layer. The results further demonstrated that lncRNAs might be involved in the biological processes by modulating gene expression. Taken together, this study provides lncRNA profiles in the ovary of tongue sole and further insight into the role of lncRNA involvement in regulating reproduction in tongue sole.

## Introduction

Transcriptome sequencing is an effective method for investigating the important functions of long non-coding RNAs (lncRNAs), a kind of non-coding RNAs without protein-coding potential with lengths longer than 200 nucleotides (Quinn and Chang, 2016). LncRNAs have been studied for their function in regulating the expression of genes through various transcriptional and posttranscriptional mechanisms (Dykes and Emanueli, 2017) such as epigenetic modification (Mercer and Mattick, 2013), chromatin remodeling (Tsai et al., 2010), repression or activation of translation (Carrieri et al., 2012) and miRNA sponging (Faghihi et al., 2010). With more sensitive sequencing technologies, a steadily increasing number of studies have shown that lncRNAs play critical roles in various biological processes including cell differentiation and development (Fatica and Bozzoni, 2014), immune responses (Chen et al., 2017), and diseases (Kopp, 2019). These studies concerning the identification and function of lncRNAs have mainly focused on humans and other mammals, while research in fish species, especially teleosts, which include the largest number of living species among all scientific classes of vertebrates, lags far behind that in mammals.

Reproduction, a limiting factor for aquaculture, is a complicated physiological process. In teleosts, the reproductive cycle is dominated by estradiol in oocyte growth and by progesterone in oocyte maturation prior to ovulation. Several enzymes, such as aromatase P450c19 (*cyp19a1*), cytochrome P450c17 (*cyp17a1*), and 17β-hydroxysteroid dehydrogenase type 1 (*hsd17b1*), are involved in the synthesis of key steroid hormones (Nagahama and Yamashita, 2008). P450c19 is the key enzyme that converts testosterone to estradiol. In this process, P450c17 is necessary of the steroid precursor shift that must occur prior to oocyte maturation (Nagahama and Yamashita, 2008). In addition, *hsd17b1* is an essential enzyme for converting estrone (E_1_) to active, receptor-binding estradiol (E_2_) in fish (Tokarz et al., 2015). Ovulation, a prerequisite for oocyte fertilization, is completed by follicular rupture, which requires extracellular matrix (ECM) dissolution (Ogiwara and Takahashi, 2019). The matrix metalloproteinase (MMP) system is required for the hydrolysis of ECM proteins present in the follicle layers of ovulating follicles. The nuclear progestin receptor (*pgr*), induced by luteinizing hormone, is an essential mediator of ovulation that complexed with maturation-inducing steroids in the nucleus to become an active transcription factor.

A number of studies have demonstrated that lncRNAs play a vital role in reproductive processes (Taylor et al., 2015), including sex hormone responses (Yang et al., 2013), oocyte meiosis (Yang et al., 2020), and ovulation (Lian et al., 2020). Emerging evidence from humans has identified the roles of lncRNAs in reproduction-related systems (Wang and Qin, 2019) and as key regulators of the normal development of granulosa cells (Tu et al., 2020). A novel lncRNA (lncRNA2193) that participates in oocyte meiosis and induces oocyte maturation is found in porcines (Yang et al., 2020). A previous study showed that oocyte-specific lncRNAs could control oocyte development and early embryogenesis in cattle (Wang et al., 2020). Additionally, several lncRNAs have been filtered and shown to regulate the synthesis of progesterone, oogenesis and oocyte maturation in goats (Liu Y. et al., 2018). The identification of lncRNAs involved in processes such as growth and development (Ali et al., 2018; Wu et al., 2020), stress responses (Dettleff et al., 2020; Quan et al., 2020), immune responses (Liu et al., 2019; Zheng et al., 2021), and sex differentiation (Cai et al., 2019; Feng et al., 2020) in fish species has begun to be reported in recent years. However, studies involving the systematic identification and characterization of lncRNAs or the roles of lncRNAs in tongue sole reproduction are lacking.

Tongue sole is marine flatfish that is an important aquaculture species in China with high economic value. Due to overfishing, its wild resources have decreased. A tongue sole aquaculture industry has developed in the last few years. However, there are some problems in culturing tongue sole. Its reproductive dysfunctions may be due to environmental and endogenous factors that restrict its reproduction (Shi et al., 2016; Song et al., 2020). In recent years, research on the reproduction of tongue sole has focused on the identification of candidate functional genes (Liu et al., 2016) and endocrine mechanisms. However, research on the lncRNA regulatory mechanism in the reproduction of tongue sole remains limited.

Thus, there is an urgent need to identify and characterize lncRNAs and investigate the regulatory mechanisms of oocyte growth, maturation and ovulation to improve the efficiency of reproduction in tongue sole. Therefore, in this study, three ovarian stages of tongue sole [stage IV: late vitellogenesis, stage V: maturation stage, and stage VI: after ovulation (Shi et al., 2016)] were chosen for sequencing to obtain their transcriptome profiles and investigate the potential involvement of lncRNAs in oocyte growth, maturation, and ovulation. This transcriptome sequencing analysis provides profiles that are useful for understanding the molecular regulatory mechanism of reproduction in tongue sole. The identification of the potential functions of lncRNAs provides a foundation for clearly understanding the factors that regulate oocyte growth, maturation and ovulation, which will allow the efficiency of tongue sole reproduction to be improved.

## Materials and Methods

### Sample Preparation and Ethics Statement

We described the experimental fish and the applied feeding and sampling procedures previously (Shi et al., 2016). Briefly, we collected tongue sole ovary samples from three ovarian development stages in triplicate. The identification of ovarian development stages is detailed in our previous studies (Shi et al., 2016). In addition, we collected triplicate samples of 12 tissues (brain, pituitary, liver, heart, ovary, kidney, heart kidney, muscle, stomach, testis, spleen, and intestines) of mature tongue sole. The tissues were immediately frozen in liquid nitrogen and stored in a refrigerator at −80°C to prevent degradation until being used for RNA isolation.

The collection and handling of the animals used in this study were approved by the Animal Care and Use Committee at the Chinese Academy of Fishery Sciences, and all the experimental procedures were performed in accordance with the guidelines for the Care and Use of Laboratory Animals at the Chinese Academy of Fishery Sciences. No endangered or protected species were involved in this experiment.

### RNA Isolation, Library Preparation, and RNA Sequencing

Total RNA was extracted using Total RNA Extraction Reagent (Vazyme Biotech, China) according to the manufacturer’s recommendations. The concentration and purity of the RNA were evaluated with a biophotometer (OSTC, China). The quality was assessed via agarose gel electrophoresis. After total RNA was extracted, rRNAs were removed by Ribo-Zero^TM^ Magnetic Kit (Epicentre, Madison, WI, United States) to retain mRNAs and ncRNAs. Then the enriched mRNA and ncRNAs were fragmented into short fragments by using fragmentation buffer and reverse transcribed into complementary DNA (cDNA) with random primers from RNA Library Prep Kit (NEB, United States). After second-strand cDNA synthesis, the cDNA fragments were purified with a QiaQuick PCR extraction kit; they were then subjected to end repair, poly (A) addition, and Illumina sequencing adapter ligation. After the digestion of second-strand cDNA by uracil-N-glycosylase (UNG), the products were size selected by agarose gel electrophoresis, PCR amplified, and sequenced using an Illumina HiSeqTM 4000 system by Gene *Denovo* Biotechnology Co. (Guangzhou, China).

### Sequence Data Processing and Analysis

To obtain high-quality clean reads, reads were further filtered by fastp (version 0.18.0) (Chen et al., 2018). Raw data containing adapters or low-quality (*q*-value ≤ 20) bases were removed from the subsequent analysis. Briefly, adapter sequences were removed according to the setting of retaining reads longer than 50 bp. Reads consisting of all “A” bases or containing more than 10% unknown nucleotides (N) were also removed. Reads were mapped to the rRNA database with Bowtie2 (2.2.8) (Langmead and Salzberg, 2012). After removing mapped rRNA reads, the remaining reads were aligned to the tongue sole reference genome by using TopHat2 (version 2.1.1) (Kim et al., 2013). Transcript reconstruction was carried out with Cufflinks (Trapnell et al., 2012), TopHat2 and Cuffmerge software. We used Cuffcompare to identify new transcripts with lengths greater than 200 bp, and more than 2 exons. We used the Coding-Non-Coding-Index (CNCI, version 2) (Sun et al., 2013) and Coding Potential Calculator (CPC^1^) (Kong et al., 2007) and the protein database SwissProt to predict the coding capacity of the new transcripts. The transcriptional intersections without coding potential and protein annotation information were defined as lncRNAs. According to their locations relative to protein coding genes, the lncRNAs were classified into five types: intergenic lncRNAs, bidirectional lncRNAs, intronic lncRNAs, antisense lncRNAs, and sense-overlapping lncRNAs.

### Differentially Expressed Transcripts and Functional Enrichment Analysis

RSEM software (Li and Dewey, 2011) was used to quantify transcript abundances. The expression levels of all transcripts were normalized using the fragments per kilobase of transcript per million mapped reads (FPKM) method. The edgeR package^2^ was used to identify significant differentially expressed (DE) mRNAs and lncRNAs among groups showing a false discovery rate (FDR) < 0.05 and fold change (FC) ≥ 2. All DE genes were mapped to GO terms in the Gene Ontology database^3^, gene numbers were calculated for every term, significantly enriched GO terms in DEGs comparing to the genome background were defined by hypergeometric test. Kyoto Encyclopedia of Genes and Genomes (KEGG) pathway enrichment analysis identified significantly enriched metabolic pathways or signal transduction pathways in DE genes comparing with the whole genome background using a hypergeometric test. A *p*-value < 0.05 was considered to indicate significant enrichment. Both GO and KEGG analyses were conducted by Gene *Denovo* Biotechnology Co. (Guangzhou, China).

### Gene Set Enrichment Analysis

Gene set enrichment analysis (GSEA) is a powerful and flexible analytical method that determines whether a previously defined set of genes, rather than a single gene, shows statistically significant, concordant differences between two biological states (Subramanian et al., 2005). Traditional enrichment analysis methods, such as GO and KEGG analyses, have focused on whether a group of DE genes is enriched for a pathway or ontology term by using overlap statistics such as the cumulative hypergeometric distribution. However, GSEA considers all genes, not only those that are significantly DE. In GSEA, significance is assessed by permuting the class labels, which preserves gene-gene correlations and thus provides a more accurate null model. The analysis was performed using GSEA software^4^ and MSigDB to identify whether a set of genes associated with specific GO terms or pathways showed significant differences between the two groups. Briefly, we input a gene expression matrix and ranked genes via the signal-to-noise normalization method. Enrichment scores and *p*-values were calculated with default parameters. In this study, we used the following threshold criteria to obtain the significantly enriched pathways: significant *p*-value, *p* < 0.05; significant *q*-value, *q* < 0.25; and normalized enrichment score (| NES|) > 1.

### Association Analysis of lncRNA-mRNA

The target genes of lncRNA transcripts were predicted through *antisense*, *cis*-, and *trans*-regulation analysis. Some *antisense* lncRNAs may regulate gene silencing, transcription and mRNA stability. To reveal the interaction between *antisense* lncRNAs and mRNAs, RNAplex software^5^ (Tafer and Hofacker, 2008) was used to predict the complementary correlations of antisense lncRNAs and mRNAs. One of the functions of lncRNAs is the *cis*-regulation of their neighboring genes in the same allele. Upstream lncRNAs showing the intersection of promoters or other *cis*-elements may regulate gene expression at the transcriptional or posttranscriptional level. Downstream or 3′UTR lncRNAs may have other regulatory functions. Thus, lncRNAs that had been previously annotated as “unknown regions” were annotated again. LncRNAs located less than 100 kb upstream/downstream of a gene are likely *cis*-regulators. On the other hand, lncRNAs can have an effect on remote target genes. We analyzed the correlation of expression between lncRNAs and protein-coding genes to identify target genes of lncRNAs showing *p* ≥ 0.9. Cytoscape (Shannon et al., 2003) software (v3.6.0) was used to analyze and visualize the interaction network relationships.

### Construction of a Competing Endogenous RNA Network

The competing endogenous RNA (ceRNA) network was constructed based on ceRNA theory. The mRNA-miRNA and lncRNA-miRNA expression correlations were evaluated using the Spearman rank correlation coefficient (SCC). Pairs with an SCC < −0.7 were selected as negatively coexpressed lncRNA–miRNA pairs or mRNA-miRNA pairs. The expression correlation between lncRNAs and mRNAs was evaluated using the Pearson correlation coefficient (PCC). Pairs with a PCC > 0.9 were selected as coexpressed lncRNA–mRNA pairs, and both the mRNA and lncRNA in each pair were targeted and negatively coexpressed with a common miRNA. We used a hypergeometric cumulative distribution function test to determine whether the common miRNA sponges between the two genes were significant. As a result, only the gene pairs with a *p*-value of less than 0.05 were selected. Cytoscape software (v3.6.0) was used to analyze and visualize the interaction network relationships.

### Validation by Real-Time Quantitative PCR

Eight transcripts, including four mRNAs and four lncRNAs, were selected to validate the RNA sequencing (RNA-Seq) results using real-time quantitative PCR (RT-qPCR). Gene-specific primers were designed with Primer 5 software (Premier Biosoft International). The β*-2-m* gene was used as the endogenous control for normalization, and all primers are shown in Supplementary Table 1. First-strand cDNA was synthesized with HiScript III RT SuperMix for qPCR (+gDNA wiper) (Vazyme Biotech, China) according to the manufacturer’s instructions. The 20 μL RT-qPCR reaction mixture consisted of 2 μL of cDNA template, 0.4 μL of both primers, 10 μL of ChamQ SYBR Color qPCR Master Mix (2×), 0.4 μL of cDNA and 6.8 μL of RNase-free water. PCR amplification was performed as follows: incubation in a 96-well optical plate at 95°C for 30 s, followed by 40 cycles of 95°C for 10 s and 60°C for 30 s. Melting curves were also plotted (60–90°C) to ensure that a single PCR product was amplified for each pair of primers. All reactions were carried out in a StepOne Plus Real-Time PCR system (Applied Biosystems). The experiments were carried out in triplicate for each data point. The relative quantification of mRNA and lncRNA transcript expression was performed using the 2^−ΔΔCt^ method (Livak and Schmittgen, 2001).

### Tissue Distribution of Candidate lncRNAs by RT-qPCR

Real-time quantitative PCR was used to determine lncRNA expression levels in 12 tissues (brain, pituitary, ovary, liver, testis, kidney, head kidney, heart, spleen, muscle, stomach, and intestines). We chose two lncRNAs (XR_522278.2 and XR_522171.2) with a potential regulatory relationship with the *cyp17a1* and *cyp19a1* genes. Briefly, three parallel samples of different tissues from three adult tongue sole individuals were prepared for RNA isolation. The total RNA of the 12 tissues was reverse transcribed into cDNA. The methods of reverse transcription and RT-qPCR have been mentioned above. The lncRNA primers are listed in Supplementary Table 1.

### Dual-Fluorescence *in situ* Hybridization of lncRNAs and mRNAs

To identify the regulation of target genes by lncRNAs, we performed a dual-fluorescence *in situ* hybridization (ISH) assay, which was modified with reference to previous studies (Qi et al., 2017). First, stage V ovary tissues of tongue sole were collected and fixed in 4% paraformaldehyde in phosphate-buffered saline (PBS) for 24 h and then preserved in paraffin. The sections for the ISH were 7 mm thick. In dual-fluorescence ISH, the probes for lncRNAs (XR_522278.2 and XR_522171.2) and mRNAs (*cyp17a1* and *cyp19a1*) were labeled with DIG and biotin (Roche Diagnostics, Mannheim, Germany), respectively. The sections were washed with PBS and blocked with blocking buffer (10% goat serum, Invitrogen, United States) after hybridization and posthybridization. Next, the sections were incubated with a peroxidase-conjugated anti-DIG secondary antibody (diluted 1:500 with blocking buffer, Roche Diagnostics, Mannheim, Germany) for 1 or 2 h. After two washes with PBS for 5 min each time, tyramide kits with Alexa Fluor 488 (Invitrogen, United States) were used for chromogenic reaction for 30 min. A fluorescence microscope (Olympus BX53F, Japan) was used to observe that the first round of staining was complete and that the samples were ready for the second round of fluorescein detection. The sections were incubated with 3% H_2_O_2_ for 20 min to remove HRP conjugated to the antibiotin, followed by two washes with PBS for 5 min each time. The sections were incubated with a peroxidase-conjugated streptavidin secondary antibody (Proteintech, United States) for 1 or 2 h. After washing similar to the previous washing step, tyramide kits with Alexa Fluor 594 (Invitrogen, United States) were used for the second chromogenic reaction for 30 min. Incubation was stopped by washing with PBS when the signal was detected. The sections were stained with DAPI (10 μg/mL, Solarbio, China) and then covered with coverslips with antifade mounting medium (Beyotime, China). Photos were obtained with a fluorescence microscope (Olympus, Japan).

## Results

### Overview of RNA-Seq

To investigate the ovary transcriptome mechanism and identify the role of lncRNAs in the reproduction of tongue sole, nine cDNA libraries were constructed and sequenced, resulting in a total of 730.8 million (730,797,538) clean reads after primary filtering. After filtering out the low-quality reads, 720.5 billion (720,475,082, 98.59%) high-quality clean reads were further processed. On average, 99.84% of the high-quality reads were not mapped to the rRNA database. Among the unmapped reads, 78.35% were successfully mapped to the reference genome. Approximately 77.69% of the uniquely mapped reads were used for transcript construction (Supplementary Table 2).

**FIGURE 1:**
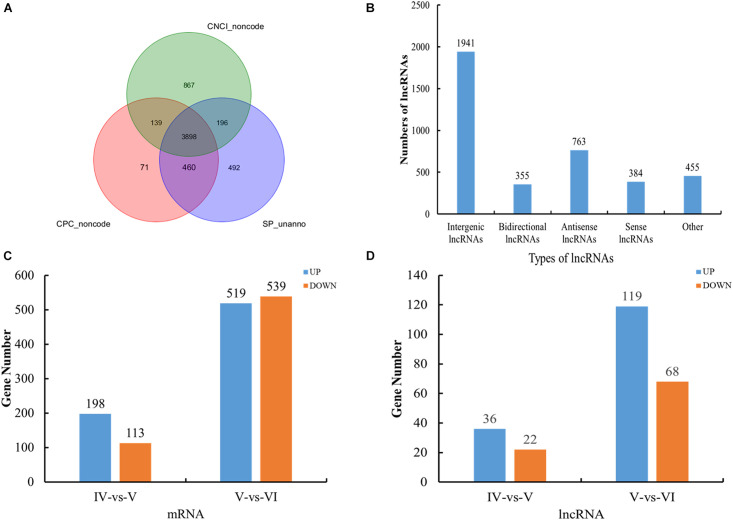
Overview of RNA sequencing in the tongue sole ovary. **(A)** Venn diagrams of annotated results of CPC, CNCI, and SwissProt. **(B)** The categories of novel lncRNAs transcripts. **(C)** The histogram of differently expressed mRNA statistics. **(D)** The histogram of differently expressed lncRNA statistics.

According to the location of the assembled transcripts in the reference genome, the novel transcripts were filtered according to the following criteria: transcript length ≥ 200 bp and exon number ≥ 2. We considered the new transcripts without coding capacity and protein annotation information to be lncRNAs (Figure 1A). A total of 353,809 mRNAs (218,325 known and 135,484 novel) and 33,562 lncRNAs (10,201 known and 23,361 novel) were obtained. The detailed genomic information and functional annotations of the new transcripts are shown in Supplementary Table 3.

The novel lncRNA transcripts could be divided into five categories. We detected 1,941 intergenic lncRNAs, 355 bidirectional lncRNAs, 763 antisense lncRNAs, 384 sense-overlapping lncRNAs, and 455 other lncRNAs. There were no intronic lncRNAs (Figure 1B).

### Analysis of Differentially Expressed mRNAs and lncRNAs

**FIGURE 2:**
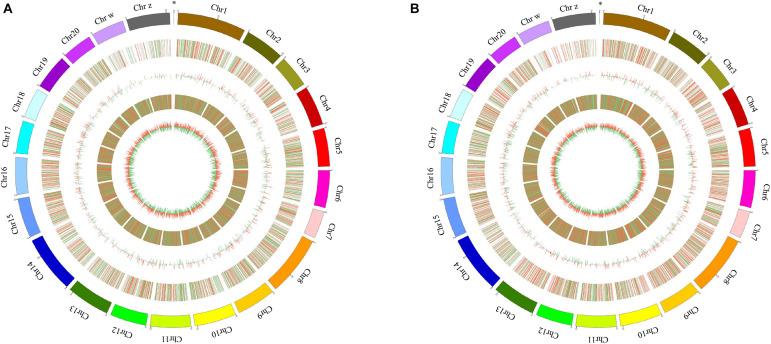
Circos plot diagram of differential expressed (DE) lncRNA and mRNA in stage IV vs. V **(A)** and stage V vs. VI **(B)**. The outermost circle is the autosomal distribution of tongue sole; the second circle is the DE lncRNA on the chromosome, the red line represents up regulation, the green line represents down-regulation; the third circle is the histogram of DE lncRNAs at different positions. Red represents up regulation, green represents down regulation, and the higher column, indicates the more DE gene numbers. The fourth circle is the distribution of DE mRNAs on the chromosome, and the color distribution is the same as lncRNA; the innermost circle is the column with DE mRNAs at different positions, color distribution is the same as lncRNA. The “^∗^” represents mitochondrion genome.

A total of 311 and 1,058 mRNAs were DE in stage IV vs. V and V vs. VI, respectively, and 79 mRNAs were DE in both comparison groups (Supplementary Table 4). Among these DE mRNAs, 198 upregulated and 113 downregulated mRNAs were identified in stage IV vs. V, and 519 upregulated and 539 downregulated mRNAs were identified in stage IV vs. V (Figure 1C). A total of 58 and 187 lncRNAs were DE in stage IV vs. V and V vs. VI, respectively, and 13 lncRNAs were DE in both comparison groups (Supplementary Table 4). Among these DE lncRNAs, 36 upregulated and 22 downregulated lncRNAs were identified in stage IV vs. V, and 119 upregulated and 68 downregulated lncRNAs were identified in stage V vs. VI (Figure 1D). Circos software was used to visualize the DE mRNAs and lncRNAs. The DE mRNAs and lncRNAs in the two comparison groups (stage IV vs. V and stage V vs. VI) are shown in Figures 2A,B.

### GO and KEGG Pathway Enrichment Analysis of DE mRNAs

**FIGURE 3:**
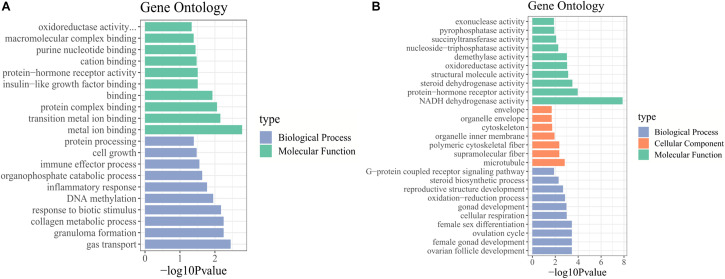
GO enrichment analysis of DE mRNAs. GO terms enriched in **(A)** stage IV vs. V. and **(B)** stage V vs. VI.

We identified the biological functions of DE mRNAs through GO and KEGG pathway analysis. The GO terms with *q*-values ≤ 0.05 were considered significantly enriched (Supplementary Table 5). In stages IV vs. V, the identified molecular functions consisted mainly of binding functions, including metal ion binding, protein complex binding and purine nucleotide binding. Among biological processes, the significantly enriched GO terms included cell growth, collagen metabolic process, and DNA methylation (Figure 3A). In stage V vs. VI, the cellular component terms consisted of the cytoskeleton and microtubules. The most important molecular functions were related to steroid dehydrogenase activity, oxidoreductase activity, and protein hormone receptor activity. Among biological processes, the significantly enriched GO terms included steroid biosynthetic process, ovarian follicle development, reproductive structure development, and ovulation cycle process (Figure 3B).

**FIGURE 4:**
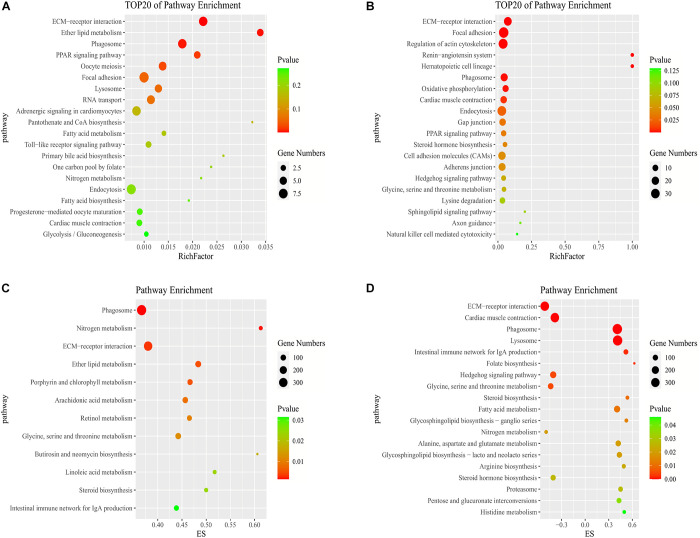
Pathways enrichment analysis of DE mRNAs. KEGG pathway enriched in stage IV vs. V **(A)** and stage V vs. VI **(B)**. GSEA of KEGG pathway enriched in ovarian stage IV vs. V **(C)** and stage V vs. VI **(D)**.

In the KEGG pathway analysis, we used a *q*-value ≤ 0.05 as a threshold, and a total of 6 and 14 pathways were found to be significantly enriched in stage IV vs. V and V vs. VI, respectively (Supplementary Table 5). We selected the top 20 pathways in the KEGG enrichment analysis of stage IV vs. V and V vs. VI (Figures 4A,B). Among these pathways, the ECM-receptor interaction, PPAR signaling pathway, phagosome and focal adhesion pathways, which are associated with signaling molecules and interactions, the endocrine system, transport and catabolism and the cellular community, were significantly enriched in both comparison groups. Several genes were involved in the above pathways, such as thrombospondin-1 (*thbs1*), fibronectin-like (*fn1*), angiopoietin-related protein 4 (*angptl4*), cathepsin S (*ctss*), tenascin (*tnc*), and phosphoinositide 3-kinase regulatory subunit 5 (pik3r5) (Supplementary Table 6). In stages IV vs. V, oocyte meiosis was significantly enriched, which is related to oocyte development and maturation processes. Several genes, such as ribosomal protein S6 kinase alpha-1 (*rps6ka1*), serine/threonine-protein phosphatase 2 (*ppp2r5c*), F-box only protein 43 isoform X4 (*fbxo43*), and serine/threonine-protein kinase isoform X2 (*slk*), were involved in this pathway. In stage V vs. VI, several cellular process-related pathways were identified, including the regulation of the actin cytoskeleton, adherens junction and endocytosis pathways, which are associated with cell motility, proliferation, and catabolism. The steroid hormone biosynthesis pathway was also significantly enriched and included several genes, such as *cyp19a1, cyp17a1*, and *hsd17b1* (Supplementary Table 6).

According to the functional enrichment analysis and a literature search, a total of 100 candidate DE genes related to the reproductive cycle were filtered and divided into six categories by function, including signal transduction, metabolism, immune response, cell junction, transport and catabolism, and cell growth and death categories (Supplementary Table 7).

### GSEA

To further study the biological functions of mRNAs and identify more undiscovered but very important pathways, we carried out GSEA. Generally, the gene sets were considered significant according to the following criteria: | NES| > 1, NOM *p*-value < 0.05, and FDR *q*-value < 0.25 (Supplementary Table 8). In stages IV vs. V, the identified cellular components were related to cell junctions. The molecular functions consisted of growth factor binding. In the biological process category, the regulation of cell development and differentiation was identified. In stage V vs. VI, the molecular functions consisted of oxidoreductase activity, and the biological processes consisted of muscle contraction and blood circulation (Supplementary Table 8). A total of 12 and 19 pathways were observed by GSEA to be significantly enriched in stage IV vs. V and V vs. VI, respectively (Figures 4C,D). Some pathways that we described above were also enriched according to GSEA, such as the ECM-receptor interaction and phagosome and steroid hormone biosynthesis pathways. In addition, steroid biosynthesis was significantly enriched in both stage IV vs. V and V vs. VI. The enrichment scores of the steroid biosynthesis pathway in the two comparison groups were driven by 16 and 13 genes, respectively. Some of these core genes were not significantly DE genes but contributed to significant pathway enrichment, such as 1,25-dihydroxyvitamin D (Mercer and Mattick, 2013) 24-hydroxylase (*cyp24a1*), sterol O-acyltransferase 1 (*soat1*), and delta (Yang et al., 2013)-sterol reductase (*tm7sf2*) (Supplementary Table 8). Furthermore, metabolic pathways such as glycine, serine and threonine metabolism, retinol metabolism and fatty acid metabolism were enriched according to GSEA.

### Identification and Enrichment Analysis of Target Genes of lncRNAs

**FIGURE 5:**
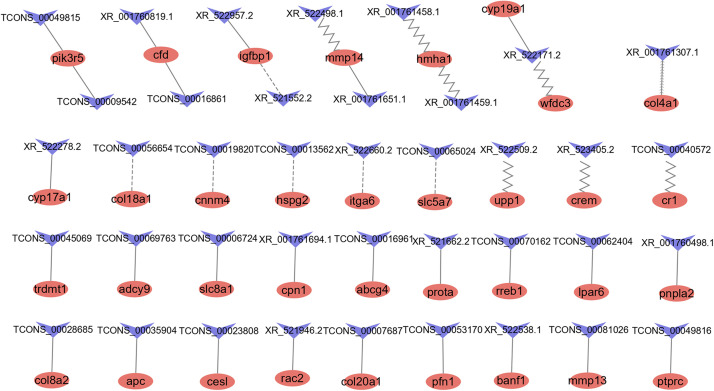
LncRNAs and their potential *antisense, cis*-, and *trans-*target genes. Red ellipse represents mRNAs, blue “V” represents lncRNAs. The contiguous arrow, zigzag, dash, and solid line indicates the interaction between DE lncRNAs and its *antisense, cis-*, and *trans- positive* and *negative* target genes.

To predict the possible interaction of DE lncRNAs and mRNAs, we conducted *antisense, cis-*, and *trans*-regulatory relationship analyses. According to previous studies, lncRNAs can regulate gene expression through *antisense* and *cis-*regulatory mechanisms. These lncRNAs show relationships with genes according to their physical positions. In addition, lncRNAs can affect mRNAs that are not adjacent to the site of their transcription via *trans*-regulatory mechanisms. The Cytoscape platform was used to visualize these correlations (Figure 5).

#### Antisense Regulatory Relationship Analysis

A total of 1,767 pairs of *antisense* regulatory relationships were observed (Supplementary Table 9). Among these relationships, five lncRNAs exhibited *antisense* complementary correlations with four DE mRNAs, as shown in Supplementary Table 7. Among these genes, only one (*col4a1*) was an *antisense* target of a DE lncRNA (XR_001761307.1). Enrichment analysis indicated that *antisense* target genes were significantly enriched in 16 cellular component GO terms, 38 molecular function GO terms, 35 biological process GO terms, and 11 KEGG pathways (Supplementary Table 10).

#### Cis-Regulatory Relationship Analysis

A total of 6,019 pairs of *cis*-regulatory relationships were observed (Supplementary Table 9). Among these relationships, we identified 23 lncRNAs showing potential *cis*-target regulatory relationships with 14 DE mRNAs, as listed in Supplementary Table 7. Among these lncRNAs, only eight were DE. The *hmha1* gene was a potential *cis*-target gene of eight lncRNAs. Enrichment analysis indicated that *cis*-target genes were significantly enriched in 16 cellular component GO terms, 29 molecular function GO terms and 90 biological process GO terms and 15 KEGG pathways (Supplementary Table 10).

#### Trans Regulatory Relationship Analysis

We identified 5,359 coexpression pairs of lncRNAs and DE mRNAs (Supplementary Table 9). Of these, we identified 44 coexpression pairs of lncRNAs and mRNAs listed in Supplementary Table 7. Among these, there were 31 coexpression pairs of DE lncRNAs and DE mRNAs, 25 pairs exhibited a positive correlation, and six pairs exhibited a negative correlation. Enrichment analysis indicated that trans-target genes were significantly enriched for nine cellular component GO terms, 27 molecular function GO terms, and 93 biological process GO terms and 15 KEGG pathways (Supplementary Table 10).

The top 20 pathways identified in the KEGG enrichment analysis of the three types of regulatory relationships are shown in Supplementary Figures 1A–C. The pathways related to the cell cycle, signaling and reproduction included apoptosis, the TGF-beta pathway, the Wnt signaling pathway, the ErbB signaling pathway, the MAPK signaling pathway, the GnRH signaling pathway, and progesterone-mediated oocyte maturation. These results indicated that these lncRNAs may potentially play a role in reproduction.

### mRNA-miRNA-lncRNA Interaction Analysis

We identified mRNAs and lncRNAs with an FC ≥ 2 and an FDR < 0.05 in comparisons as significant DE transcripts and miRNAs with an FC ≥ 2 and *p*-value < 0.05. Based on the DE mRNA, lncRNA and miRNA data, three software programs, MIREAP, miRanda, and TargetScan, were used to predict the targets of each miRNA. A ceRNA network was constructed after obtaining the mRNA-miRNA and lncRNA-miRNA pairs, in which the mRNA and lncRNA in each pair were targeted and negatively coexpressed with a common miRNA (Supplementary Table 11).

**FIGURE 6:**
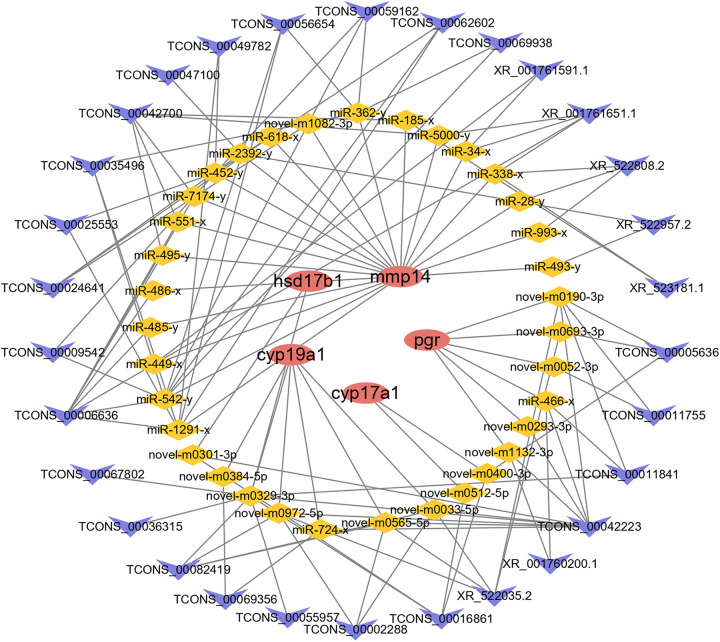
The ceRNA network of *cyp17a1*, *cyp19a1*, *mmp14*, *pgr*, and *hsd17b1.* Red ellipse represents mRNAs, yellow diamond represents miRNAs, and blue “V” represents lncRNAs.

The interactions between lncRNAs and DE mRNAs predicted through ceRNA network analysis are listed in Supplementary Table 1 (Supplementary Figure 3). The network consisted of 164 lncRNAs, 302 miRNAs, and 63 mRNAs. On the basis of combined ceRNA network and pathway enrichment analyses, five reproduction-related genes, *cyp17a1, cyp19a1, mmp14, pgr*, and *hsd17b1*, were selected to predict their interactions with lncRNAs. Thirty lncRNAs interacted with four mRNAs by competing for 35 miRNAs (Figure 6). Enrichment analysis was carried out to analyze the functions of the mRNAs involved in the ceRNA network. A *p*-value < 0.05 was set as the threshold for significantly enriched GO terms and pathways. A total of 27 cellular component GO terms, 37 molecular function GO terms and 101 biological process GO terms were significantly enriched. In addition, a total of 27 pathways were significantly enriched, including reproduction-related pathways such as the GnRH signaling pathway and steroid hormone biosynthesis pathways (Supplementary Figure 2 and Supplementary Table 12).

### Validation by RT-qPCR

To validate the RNA-Seq results, eight transcripts, including four DE mRNAs and four DE lncRNAs, were selected for RT-qPCR. The results showed that the expression trends for all four DE mRNAs and four DE lncRNAs were consistent with the RNA-Seq results, with *R*^2^ = 0.9472 and *R*^2^ = 0.8675, respectively. Generally, the RNA-Seq results were mostly confirmed by qPCR analysis, implying the reliability and accuracy of the RNA-Seq analysis (Supplementary Figure 4).

### Analysis of Expression Levels of Candidate lncRNAs in the Ovary

**FIGURE 7:**
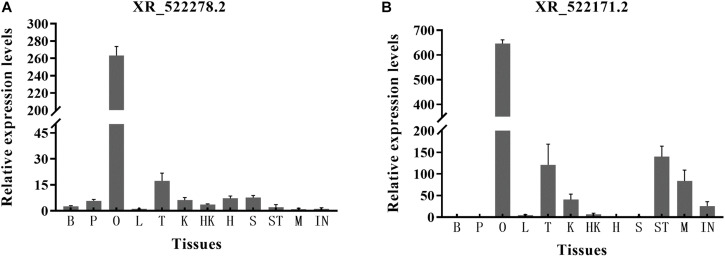
The expression level of lncRNAs XR_522278.2 **(A)** and XR_522171.2 **(B)** in 12 tissues. B, brain; P, pituitary; O, ovary; L, liver; T, testis; K, kidney; HK, head kidney; H, heart; S, spleen; ST, stomach; M, muscle; IN, intestines.

For the two lncRNAs (XR_522278.2 and XR_522171.2) showing a potential regulatory relationship with the *cyp17a1* and *cyp19a1* genes, respectively, we explored the expression distribution among 12 tissues of tongue sole by RT-qPCR. The lncRNAs were widely expressed in the 12 tissues but showed the highest expression in the ovary. The highest expression level of lncRNA XR_522278.2 was detected in the ovary (263.3-fold relative to control). The highest expression level of lncRNA XR_522171.2 was also detected in the ovary (646.3-fold relative to control) (Figure 7).

### Colocalization of lncRNA and mRNA in Ovarian Tissue

**FIGURE 8:**
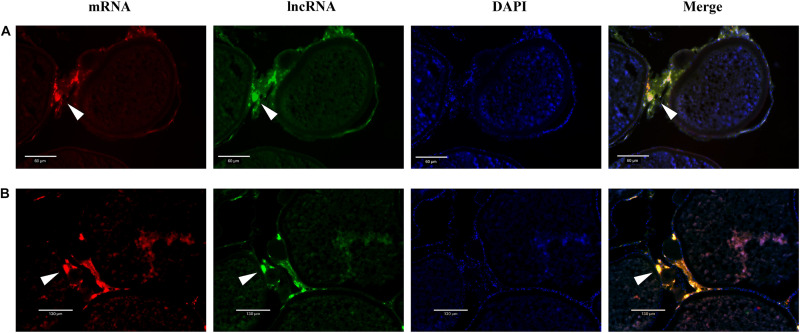
The colocalization of lncRNA and mRNA. **(A)** The colocalization of cyp17a1 (red, stained with Alexa Fluor 594) and XR_522278.2 (green, stained with Alexa Fluor 488). **(B)** The colocalization of cyp19a1 (red, stained with Alexa Fluor 594) and XR_522171.2 (green, stained with Alexa Fluor 488).

Dual-fluorescence ISH of lncRNAs and mRNAs in stage V ovaries was performed to demonstrate the potential regulation of mRNAs by lncRNAs. As shown in Figure 8, positive XR_522278.2 and *cyp17a1* signals were present in the follicular cell layer, where *cyp17a1* plays a role in the shift in steroid precursor production. Similarly, positive XR_522171.2 and *cyp19a1* signals were present in the follicular cell layer, where cyp19a1 converts testosterone to E_2_ (Figure 8).

## Discussion

Within the reproductive cycle, oocyte growth along with oocyte maturation and ovulation is a prerequisite for successful reproduction, which is important in the aquaculture industry (Das et al., 2017). To provide a comprehensive view of the changes in transcriptome levels during tongue sole ovary growth, maturation, and ovulation, RNA-Seq analysis was performed to investigate the regulatory mechanism involved and detect key gene functions and the corresponding regulatory lncRNAs. In this study, we identified 1,059 and 312 DE mRNAs in stage IV vs. V and V vs. VI, and total of 58 and 187 DE lncRNAs were observed in stage IV vs. V and V vs. VI, respectively.

It has been proven that steroid hormones are involved in the regulation of a variety of processes, such as embryonic development, sex differentiation, metabolism, immune responses, circadian rhythms, stress responses, and reproduction in vertebrates, including teleosts (Zhou et al., 2012). In the present study, we identified several DE genes that encode crucial enzymes in the biological synthesis of estradiol and progesterone, such as *cyp17a1*, *cyp19a1*, and *hsd17b1* (Tokarz et al., 2015). All of them were mainly expressed in stage V, when oocytes matured, and showed a significant decrease from stage V to VI. Moreover, the steroid hormone biosynthesis pathway was significantly enriched in stage V vs. VI, while GSEA showed that this pathway was downregulated in stage V vs. VI and that *cyp17a1*, *cyp19a1*, and *hsd17b1* were core genes in this pathway. These findings indicated that these genes and this pathway play key roles in stage V when oocytes mature.

Oocyte meiosis is an essential part of oocyte maturation in both mammals and teleosts (Nagahama and Yamashita, 2008; Zhao et al., 2020). This pathway, several genes involved, such as *fbxo43*, *slk*, and *rps6ka1*, were significantly enriched in stage IV vs. V. Early mitotic inhibitor 2 (EMI2), encoded by *fbxo43*, is a member of the F-box protein family that plays an important role in cytostatic factor arrest in *Xenopus* eggs (Tung et al., 2005; Vadhan et al., 2020). In the present study, *fbxo43* was found to be significantly DE in stage IV vs. V.

During the reproductive cycle, the ECM has been proven to participate in cell cycle processes during ovarian remodeling, ranging from follicular development and involution after ovulation to cell proliferation and differentiation (Curry and Osteen, 2003). The MMPs family is the major enzyme family responsible for ECM remodeling. Many studies in medaka have shown that MMPs participate in final ovulation, including oocyte maturation and follicle regression (Ogiwara et al., 2005; Takahashi et al., 2013). *Mmp14* is a membrane-type MMPs located at the cell surface (Pedersen et al., 2015). In this study, we found that *mmp14* was DE and exhibited the highest expression in stage VI in the ovary, after ovulation. Fibronectin and laminin are ECM proteins synthesized by follicular cells (Zhao and Luck, 1995; Yasuda et al., 2005). A study in *Pampus argenteus* identified laminin β2 as one of the main component of the basement membrane in ovarian follicles. Fibronectin is also detected in postovulatory follicular cells in *P. argenteus* (Thomé et al., 2010). In the present study, we found that *lamc3* exhibited the highest expression in stage IV but was significantly downregulated in stages V and VI. Fibronectin-like (*fn1*) increased from stage IV to stage V ovaries and reached a peak in stage VI in the ovary, when ovulation occurred. We also detected other cell adhesion-related genes, including *col4a1*, *ecm1*, *itga6*, *krt18*, and *tnc*, that were significantly DE in the ovary. Furthermore, ECM-receptor interaction and focal adhesion pathways were enriched in both stage IV vs. V and stage V vs. VI, and these pathways involved the greatest numbers of the above DE genes. These findings indicated that although endocrine hormones are necessary for the reproduction of tongue sole, the ECM is involved in all reproductive processes, especially ovulation, when follicular rupture causes the dissolution of the ECM of the follicular wall.

Some metabolic pathways were enriched in the two comparison groups according to GSEA, including retinol metabolism and fatty acid metabolism. Retinol plays an important role in mammalian placental and embryonic development (Marceau et al., 2007). Retinoic acid, a form of vitamin A, supports both male and female reproduction and embryonic development. In teleosts, retinotic acid is the key factor controlling the initiation of meiosis (Feng et al., 2015). Fatty acids may play important roles in the induction of oocyte maturation in marine teleosts, which requires n-3 long-chain PUFAs (LC-PUFAs) as essential fatty acids (Sorbera et al., 2001). In tongue sole, fatty acids have been proven to be required for gonadal steroidogenesis. An appropriate DHA:EPA ratio can induce high expression of the follicle-stimulating hormone receptor (FSHR), steroidogenic acute regulatory protein (STAR), 17α-hydroxylase (P450c17) and 3β-hydroxysteroid dehydrogenase (3β-HSD), which are involved in steroidogenesis (Xu et al., 2017). Thus, the retinol metabolism and fatty acid metabolism pathways may be involved in oocyte maturation, including steroidogenesis and oocyte meiosis, in tongue sole.

In recent years, lncRNAs have attracted increasing attention because of their involvement in modulating gene expression (Quinn and Chang, 2016). Research on the identification and characterization of lncRNAs involved in reproduction in fish, especially in tongue sole, is far behind that in mammals. Therefore, in the present study, we first obtained comprehensive expression profiles of lncRNA transcripts in three ovarian stages of tongue sole. A total of 33,562 lncRNAs (10,201 known and 23,361 novel) were obtained through high-throughput sequencing. These DE lncRNAs may have potential reproductive functions in tongue sole.

It is well established that lncRNAs can exert regulatory effects on mRNAs (Quinn and Chang, 2016; Tan-Wong et al., 2019; Elcheva and Spiegelman, 2020). The target genes of lncRNAs can be predicted on the basis of either their physical positions, in the case of *antisense* and *cis-*regulatory mechanisms, or their coexpression relationships, in the case of *trans*-regulation. In the present study, we showed that the *mmp14 gene* is a *cis*-target gene of two lncRNAs (XR_522498.1 and XR_522499.1) and that the *col4a1* and *crem genes* are the *cis*-target genes of lncRNAs XR_523405.2 and XR_001761307.1, respectively. We found that *antisense* and *cis*-target genes were enriched in the cell cycle, in processes including apoptosis and signaling pathways such as the TGF-beta signaling pathway and the Wnt signaling pathway. Several studies have shown that the TGF-beta signaling pathway and Wnt signaling pathway are involved in a broad spectrum of cellular functions, such as proliferation, apoptosis, differentiation, migration, and embryogenesis (Motomura et al., 2014; Yu et al., 2020). TGF-beta signals can lead to avian ovary granulosa cell proliferation mediated by signals transduced by the cytoplasmic signal transducer Smad (Schmierer et al., 2003). TGF-beta is involved in maintaining oocyte meiotic arrest regulated by FSH and LH in mice (Yang et al., 2019). Recent studies revealed that the inhibitory effect of FH535 (Wnt/β-catenin inhibitor) on the Wnt signaling pathway can promote the maturation of porcine oocytes and alter gene expression *in vitro* (Shi et al., 2018). In mice, cell cycle arrest is associated with the downregulation of Wnt signaling in granulosa cells (De Cian et al., 2020).

A total of 31 coexpressed pairs of DE lncRNAs and mRNAs were identified in this study, including 25 pairs with a positive correlation and six pairs with a negative correlation. Among these genes, *Igfbp1* exhibited a positive correlation with the lncRNA XR_522957.2. Previous studies have shown that different IGF-binding proteins exhibit marked expression in the preovulation period (Knight and Glister, 2006). The *cyp17a1 gene*, which encodes a key enzyme involved in steroidogenesis, exhibited a negative correlation with lncRNA (XR_522278.2). We also showed that *trans*-target genes were enriched in the MAPK signaling pathway, which plays a role in oocyte meiosis, meiotic resumption and maturation (Liang et al., 2007). Furthermore, lncRNAs have been reported to participate in the MAPK signaling pathway (Sulayman et al., 2019). Thus, the results indicated that these lncRNAs play a role in oocyte development and ovulation in tongue sole through *antisense, cis-*, and *trans*-regulatory mechanisms.

Through the analysis of mRNA, lncRNA and miRNA interactions, a ceRNA network was constructed in which lncRNAs competed for miRNA binding with mRNAs and thereby regulated the expression of mRNAs. In previous studies, ceRNA networks have been constructed to predict the mechanisms of immune infiltration in humans (Huang et al., 2019), muscle growth and development in Japanese flounder (Wu et al., 2020) and ovarian development in broody chickens (Liu L. et al., 2018). In the present study, we also constructed a ceRNA network to predict the biological functions of lncRNAs in reproduction. Interestingly, the genes closely associated with reproductive processes, including *mmp14*, *pgr, cyp17a1, cyp19a1*, and *hsd17b1*, were indicated to potentially be modulated by 30 lncRNAs via 35 miRNAs. Among these genes, *pgr* has been reported to play an important role in oocyte development and ovulation in mammalian reproduction (Akison and Robker, 2012). In addition, *pgr* knockout zebrafish exhibit normal oocyte growth and maturation but show defects in ovulation and fail to spawn, indicating the role of *pgr* in steroid-dependent genomic pathways leading to ovulation (Zhu et al., 2015). In the present study, *pgr* was found to be a DE gene that was enriched in pathways including oocyte meiosis and progesterone-mediated oocyte maturation. Twelve lncRNAs were shown to modulate *pgr* via 10 miRNAs. Moreover, we found that progesterone-mediated oocyte maturation, oocyte meiosis and the GnRH signaling pathway were enriched in DE mRNAs involved in the ceRNA network. This provides direct evidence that these lncRNAs, acting as regulators of gene expression, can modulate the expression of genes involved in ovarian growth, maturation, and ovulation in the process of reproduction.

Real-time quantitative PCR analysis revealed that the two lncRNAs (XR_522278.2 and XR_522171.2), potentially modulating *cyp17a1* and *cyp19a1* expression, respectively, were dominantly expressed in the ovary. As expected, both the XR_522278.2 and XR_522171.2 lncRNAs colocalized with their target genes *cyp17a1* and *cyp19a1*, respectively, in the follicular cell layer, which further demonstrated that the lncRNAs may play a role in the reproduction of tongue sole by regulating gene expression. Thus, lncRNAs may play roles in oocyte development, maturation and ovulation by regulating the expression of steroidogenic enzymes, transcription factors, and growth factors, which have been proven to be the key genes involved in reproduction in tongue sole.

## Conclusion

This study first identified lncRNAs in the ovary and revealed the molecular mechanisms and pathways in the ovary involved in the reproduction of tongue sole. The enrichment analysis of DE mRNAs showed that pathways related to reproductive endocrine functions and the cell cycle drove oocyte growth, maturation, and ovulation. Several genes involved in these pathways, such as *cyp17a1*, *cyp19a1*, *mmp14*, *pgr*, and *hsd17b1*, were identified. We also identified lncRNAs that may exert regulatory effects on mRNAs related to the reproduction of tongue sole through *antisense*, *cis-* and *trans*-regulatory mechanisms. On the basis of the competitive relationships between mRNAs and lncRNAs, a ceRNA network was constructed to predict the potential mRNA targets of lncRNAs. The lncRNAs of critical genes were dominantly expressed in the ovary. Moreover, lncRNAs and their target genes were colocalized in the ovarian follicular cell layer. Taken together, the results of this study highlight the role of lncRNAs in regulating the expression of mRNAs that influence reproduction and provide deeper insight into the molecular mechanisms of reproduction in tongue sole. Moreover, further investigations involving functional analysis methods such as gene knockdown and overexpression are critical for elucidating the regulatory mechanism of lncRNAs in the fish ovary.

## Data Availability Statement

The sequencing data obtained from the RNA-seq were released to the National Center for Biotechnology Information (NCBI) Sequence Read Archive (SRA) database under the accession numbers SRR14090692, SRR14090691, SRR14090690, SRR14090714, SRR14090713, SRR14090711, SRR14090700, SRR14090699 and SRR14090698.

## Ethics Statement

The animal study was reviewed and approved by the Animal Care and Use Committee at the Chinese Academy of Fishery Sciences.

## Author Contributions

YD finished the experiment. YD and HW drafted and critically revised the manuscript. YD, LL, DZ, JL, and HW analyzed and interpreted the data. BS conceived the study and critically revised the manuscript. All authors contributed to the article and approved the submitted version.

## Conflict of Interest

The authors declare that the research was conducted in the absence of any commercial or financial relationships that could be construed as a potential conflict of interest.

## Footnotes

1 http://cpc.cbi.pku.edu.cn/

2 http://www.r-project.org/

3 http://www.geneontology.org/

4 http://www.broadinstitute.org/gsea/

5 http://www.tbi.univie.ac.at/RNA/RNAplex.1.html
