# Supplementary material for: Integrated lncRNA and mRNA Transcriptome Analyses in the Ovary of Cynoglossus semilaevis Reveal Genes and Pathways Potentially Involved in Reproduction
Source: Front Genet. 2021 May 19;12:671729. doi: 10.3389/fgene.2021.671729 (PMC8172126; doi:10.3389/fgene.2021.671729)
Supplement: Supplementary file 7 [file Table_7.DOCX]

**Supplementary Table 7.** **DE genes potentially associated with reproduction of tongue sole.**

| **Functional Classification** | **DEG ID** | **Gene Name** | **Log2(fold change)** | |
| --- | --- | --- | --- | --- |
|  |  |  | **IV vs. V** | **V vs. VI** |
| **Signal Transduction** |  |  |  |  |
|  | XM_008319170.1 | complement receptor type 1-like (*cr1*) | **1.0536** | **-1.8244** |
|  | XM_008334993.2 | cAMP-responsive element modulator-like isoform X2 (*crem*) | 1.8950 | **-3.2393** |
|  | XM_017041467.1 | sodium/calcium exchanger 1-like (*slc8a1*) | 0.6125 | **-6.1043** |
|  | XM_008321529.2 | barrier-to-autointegration factor-like (*banf1*) | -0.7921 | **2.3402** |
|  | XM_008317721.2 | insulin-like growth factor-binding protein 4 (*igfbp4*) | **1.9010** | -0.7575 |
|  | XM_008338488.2 | insulin-like growth factor-binding protein 1 (*IGFBP1*) | **2.6442** | -0.1629 |
|  | XM_008309071.2 | lysophosphatidic acid receptor 6 (*lpar6*) | 0.6561 | **-1.1828** |
|  | XM_017031586.1 | ras-responsive element-binding protein 1 isoform X (*rreb1*) | 0.0000 | **10.9634** |
|  | XM_008316228.2 | complement factor D (*cfd*) | 0.5353 | **-1.4469** |
|  | XM_017034601.1 | semaphorin-3C *sema3c*) | **8.6795** | 1.4282 |
|  | XM_008325350.2 | eukaryotic translation initiation factor 5B (*eif5b*) | **11.1695** | -1.4842 |
|  | XM_017036666.1 | flocculation protein FLO11-like isoform X1 (*tgif2*) | 2.0682 | **-11.1271** |
|  | NM_001294190.1 | follicle stimulating hormone receptor precursor (*lhcgr*) | -0.2498 | **-2.9884** |
|  | XM_008313320.2 | mitogen-activated protein kinase kinase kinase 5 isoform X3 (*map3k5*) | -5.6439 | **8.5949** |
|  | XM_017039903.1 | mitogen-activated protein kinase kinase kinase kinase 4 isoform X9 (*map4k4*) | **-10.1840** | 7.4094 |
|  | XM_008331735.2 | mitogen-activated protein kinase 15 (*mapk15*) | 0.7193 | **1.3826** |
|  | XM_017040834.1 | transcription factor AP-1 (*jun*) | 1.1534 | **-1.8499** |
|  | XM_008317630.2 | transcription factor jun-B isoform X1 (*junb*) | **1.2041** | -0.6340 |
|  | TCONS_00006403 | cAMP-dependent protein kinase catalytic subunit beta isoform X3 (*prkacb*) | 0.0000 | **10.1378** |
|  | XM_008308970.2 | progesterone receptor (*pgr*) | -0.0587 | **-1.0396** |
|  | XM_008327410.2 | progesterone receptor-like isoform X1 (*prl*) | -1.4569 | **-10.4235** |
|  | XM_017035767.1 | guanine nucleotide-binding protein G(s) subunit alpha isoform X1 (*gnas*) | **-10.1799** | 6.0589 |
|  | XM_008328380.2 | G-protein coupled receptor 183-like (*gpr183*) | 0.5610 | **1.8670** |
|  | XM_008313949.2 | follicle stimulating hormone receptor isoform X1 (*fshr*) | 0.0000 | **8.4919** |
|  | NM_001294199.1 | forkhead box protein L2 (*foxl2*) | -0.3084 | **-1.8856** |
|  | XM_008328666.2 | follistatin-related protein 1 (*fstl1*) | 0.3091 | **1.0220** |
|  | TCONS_00026546 | estrogen receptor beta-like isoform X1 (*esr2*) | 5.4075 | **-10.3144** |
|  | XM_008316773.2 | phosphoinositide 3-kinase regulatory subunit 5 isoform X1 (*pik3r5*) | 0.3870 | **1.6787** |
|  | XM_017038207.1 | tyrosine-protein phosphatase non-receptor type 6 (*ptpn6*) | 1.1456 | **2.1091** |
|  | XM_017031310.1 | platelet-derived growth factor receptor alpha isoform X1 (*pdgfra*) | 0.0813 | **-1.3186** |
|  | XM_008333843.2 | angiopoietin-related protein 4-like (*angptl4*) | **2.6020** | **-1.3577** |
|  | XM_008335834.2 | bone morphogenetic protein 3 (*bmp3*) | 0.0000 | **7.8002** |
| **Cell growth and death** |  |  |  |  |
|  | XM_017037297.1 | STE20-like serine/threonine-protein kinase isoform X2 (*slk*) | **8.6439** | -0.1520 |
|  | XM_017039476.1 | securin isoform X2 (*secu*) | **-1.5647** | 0.9107 |
|  | XM_008313140.1 | serine/threonine-protein phosphatase 2A 56 kDa regulatory subunit gamma isoform-like isoform X3 (*ppp2r5c*) | **9.1882** | -1.1658 |
|  | XM_008312435.2 | CD82 antigen (*cd82*) | 0.2009 | **-1.7817** |
|  | XM_008324221.2 | testis development-related protein isoform X1 (*tdrp*) | -0.2529 | **1.4069** |
|  | XM_008324121.1 | F-box only protein 43 isoform X4 (*fbox43*) | **1.7155** | 0.2341 |
| **Immune response** |  |  |  |  |
|  | XM_008333341.1 | nucleophosmin-like isoform X1(*npm1* ) | 0.2887 | **-2.2555** |
|  | XM_008308750.2 | ribosomal protein S6 kinase alpha-1 isoform X1 (*prs6ka1*) | **-11.0521** | 9.2527 |
|  | XM_008323811.2 | cathepsin K (*ctsk*) | **1.5432** | **3.0502** |
|  | XM_008330566.2 | thrombospondin-1-like (*thbs1*) | **3.0990** | -0.8658 |
|  | XM_008321838.1 | thrombospondin-2 isoform X2 (*thbs2*) | 0.3089 | **-2.3853** |
|  | XM_008313781.2 | caspase-7 isoform X2 (*casp7*) | 0.0000 | **9.6853** |
|  | TCONS_00076729 | caspase-8-like (*casp8*) | -0.2741 | **-11.1737** |
|  | XM_008323813.1 | cathepsin S-like isoform X2 (*ctss*) | -0.7769 | **1.0860** |
| **Metabolism** |  |  |  |  |
|  | XM_008332445.2 | ATP-binding cassette sub-family G member 4-like isoform X3 (*abcg4*) | 6.3219 | **5.3309** |
|  | XM_008312538.2 | liver carboxylesterase 2-like isoform X1 (*cesl*) | -0.1262 | **1.8705** |
|  | XM_017036852.1 | prothymosin alpha-like isoform X2 (*prota*) | -2.1454 | **5.4182** |
|  | XM_008326169.2 | adenomatous polyposis coli protein isoform X1 (*apc*) | -8.4094 | **9.8918** |
|  | XM_008313788.2 | carboxypeptidase N catalytic chain (*cpn1*) | -0.1499 | **-1.6315** |
|  | XM_008323335.2 | uridine phosphorylase 1 isoform X1 (*upp1*) | 0.4990 | **1.7830** |
|  | NM_001294242.1 | aromatase-like (*cyp19a1*) | -0.1358 | **-4.7774** |
|  | XM_008329343.2 | 3 beta-hydroxysteroid dehydrogenase type 7 (*hsd3b7*) | **1.7040** | **-1.8727** |
|  | XM_008330027.2 | estradiol 17-beta-dehydrogenase 1 (*hsd17b1*) | -0.1249 | **-2.7589** |
|  | XM_008327720.2 | ectonucleotide pyrophosphatase/phosphodiesterase family member 6 isoform X1 (*enpp6*) | **2.9419** | -0.6578 |
|  | TCONS_00072643 | long-chain-fatty-acid--CoA ligase 6 isoform X1 (*acsl6*) | **-7.6653** | 4.8580 |
|  | XM_008329867.2 | adenylate cyclase type 9 isoform X1 (*adcy9*) | -1.7370 | **11.6999** |
|  | NM_001297587.1 | steroid 17-alpha-hydroxylase/17,20 lyase-like isoform X1 (*cyp17a1*) | 0.0507 | **-3.4842** |
|  | XM_017039678.1 | lipoprotein lipase isoform X1 (*lpl*) | -0.4750 | **-1.5101** |
|  | XM_008331692.1 | fatty acid-binding protein, heart-like (*fabp3*) | 0.8164 | **-1.1067** |
|  | XM_008314975.2 | lysosomal acid lipase/cholesteryl ester hydrolase-like (*lipa*) | -0.1228 | **2.0374** |
| **Transport and catabolism** |  |  |  |  |
|  | XM_008317690.2 | high affinity choline transporter 1-like (*slc5a7*) | 0.0956 | **-2.0000** |
|  | XM_008336293.2 | metal transporter CNNM4-like isoform X2 (*cnnm4*) | 0.0823 | **-1.3311** |
|  | XM_017043132.1 | clathrin heavy chain 1-like isoform X2 (*cltc*) | **9.1293** | **-9.1293** |
|  | XM_017041981.1 | ras-related protein Rab-24-like isoform X1 (*rab24*) | -2.4594 | **6.8826** |
|  | XM_008319129.2 | arf-GAP with coiled-coil, ANK repeat and PH domain-containing protein 3 isoform X3 (*acap3*) | 0.2815 | **1.1090** |
|  | XM_008309014.1 | EH domain-containing protein 2 isoform X2 (*ehd2*) | 1.3009 | **1.0783** |
|  | XM_008309606.2 | beta-arrestin-1 isoform X4 (*arrb1*) | 0.0000 | **8.6677** |
|  | TCONS_00077059 | signal transducing adapter molecule 1 (*stam*) | 0.0000 | **9.9561** |
|  | XM_008333195.2 | V-type proton ATPase catalytic subunit A-like (*atp6v1a*) | **-1.2820** | 0.4615 |
|  | XM_017036789.1 | stAR-related lipid transfer protein 9 isoform X2 (*stard9*) | -0.2442 | **-1.2353** |
| **Cell Junction** |  |  |  |  |
|  | XM_008318168.2 | matrix metalloproteinase-9 (*mmp9*) | **2.5086** | -0.5485 |
|  | XM_008308549.2 | collagenase 3-like (*mmp13*) | 0.2106 | **4.4610** |
|  | XM_008323136.2 | matrix metalloproteinase-14 (*mmp14*) | **1.1942** | 0.2895 |
|  | XM_008334732.2 | matrix metalloproteinase-14-like (*mmp14*) | **1.5289** | 0.0669 |
|  | XM_008331796.2 | receptor-type tyrosine-protein phosphatase C isoform X1 (*ptprc*) | 0.1463 | **1.8184** |
|  | XM_017031425.1 | collagen alpha-1(XVIII) chain-likec (*col15a1*) | 0.4621 | **-3.0257** |
|  | XM_008329029.2 | collagen alpha-1(XVIII) chain-like (*col18a1*) | -0.1443 | **-1.5255** |
|  | XM_017036118.1 | collagen alpha-1(XX) chain-like isoform X2 (*col20a1*) | **-7.8009** | -1.7370 |
|  | XM_008328686.2 | collagen alpha-1(IV) chain (*col4a1*) | 0.5702 | **-1.0221** |
|  | XM_008331908.2 | extracellular matrix protein 1-like (*ecm1*) | **1.3231** | -0.7452 |
|  | XM_008321223.2 | procollagen C-endopeptidase enhancer 2-like (*pcolce*) | 0.5715 | **-5.0894** |
|  | XM_008325580.2 | profilin-1 (*pfn1*) | 0.3033 | **1.3028** |
|  | XM_008329103.2 | fibronectin-like (*fn1*) | **1.3823** | **1.4466** |
|  | TCONS_00081943 | tenascin isoform X1 (*tnc*) | **2.7469** | **-2.6772** |
|  | TCONS_00013290 | zona pellucida sperm-binding protein 4-like (*zp4*) | **9.7593** | -1.2542 |
|  | XM_008325779.2 | laminin subunit gamma-3 (*lamc3*) | -0.9786 | **10.1421** |
|  | XM_008328276.2 | integrin alpha-6-like (*itga6*) | 0.1571 | **-3.0241** |
|  | XM_017040124.1 | integrin beta-2 (*itgb2*) | -0.4176 | **2.6063** |
|  | XM_008332029.2 | integrin beta-8 isoform X1 (*itgb8*) | 0.5152 | **-2.2334** |
|  | XM_008323177.2 | brain-specific angiogenesis inhibitor 1-associated protein 2-like (*baiap2*) | 0.4444 | **-1.0030** |
|  | XM_008318226.1 | keratin, type I cytoskeletal 18-like (*krt18*) | 0.4696 | **-1.1540** |
|  | XM_008319112.2 | basement membrane-specific heparan sulfate proteoglycan core protein isoform X9 (*hspg2*) | 0.1802 | **-3.4910** |
| **Others** |  |  |  |  |
|  | XM_008307271.2 | minor histocompatibility protein HA-1 isoform X1 (*hmha1*) | -0.2711 | **2.4000** |
|  | XM_008318704.2 | WAP four-disulfide core domain protein 2-like (*wfdc3*) | 0.2914 | **-3.3515** |
|  | XM_017031776.1 | tRNA (cytosine(38)-C(5))-methyltransferase isoform X1 (*trdmt1*) | -2.9710 | **4.1514** |
|  | XM_008316548.2 | ras-related C3 botulinum toxin substrate 2 (*rac2*) | 0.3270 | **1.6781** |
|  | XM_008312165.2 | patatin-like phospholipase domain-containing protein 2 (*pnpla2*) | **1.5619** | -0.9707 |
|  | XM_008322722.2 | gap junction alpha-9 protein (*gja9*) | **-2.3832** | -4.0000 |
