# Supplementary material for: Integrated lncRNA and mRNA Transcriptome Analyses in the Ovary of Cynoglossus semilaevis Reveal Genes and Pathways Potentially Involved in Reproduction
Source: Front Genet. 2021 May 19;12:671729. doi: 10.3389/fgene.2021.671729 (PMC8172126; doi:10.3389/fgene.2021.671729)
Supplement: Supplementary file 13 [file Data_Sheet_1.pdf]

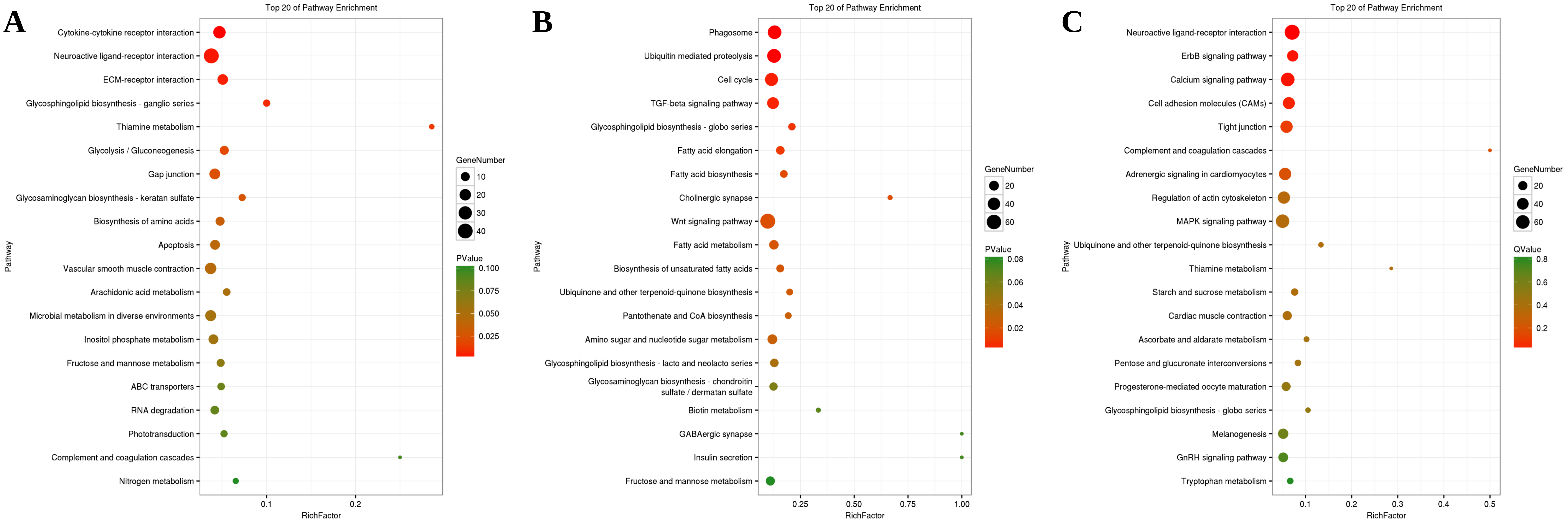

Figure S1. Top 20 significantly enriched pathways associated with *antisense* (A), *cis*- (B) and *trans*- (C) target genes.

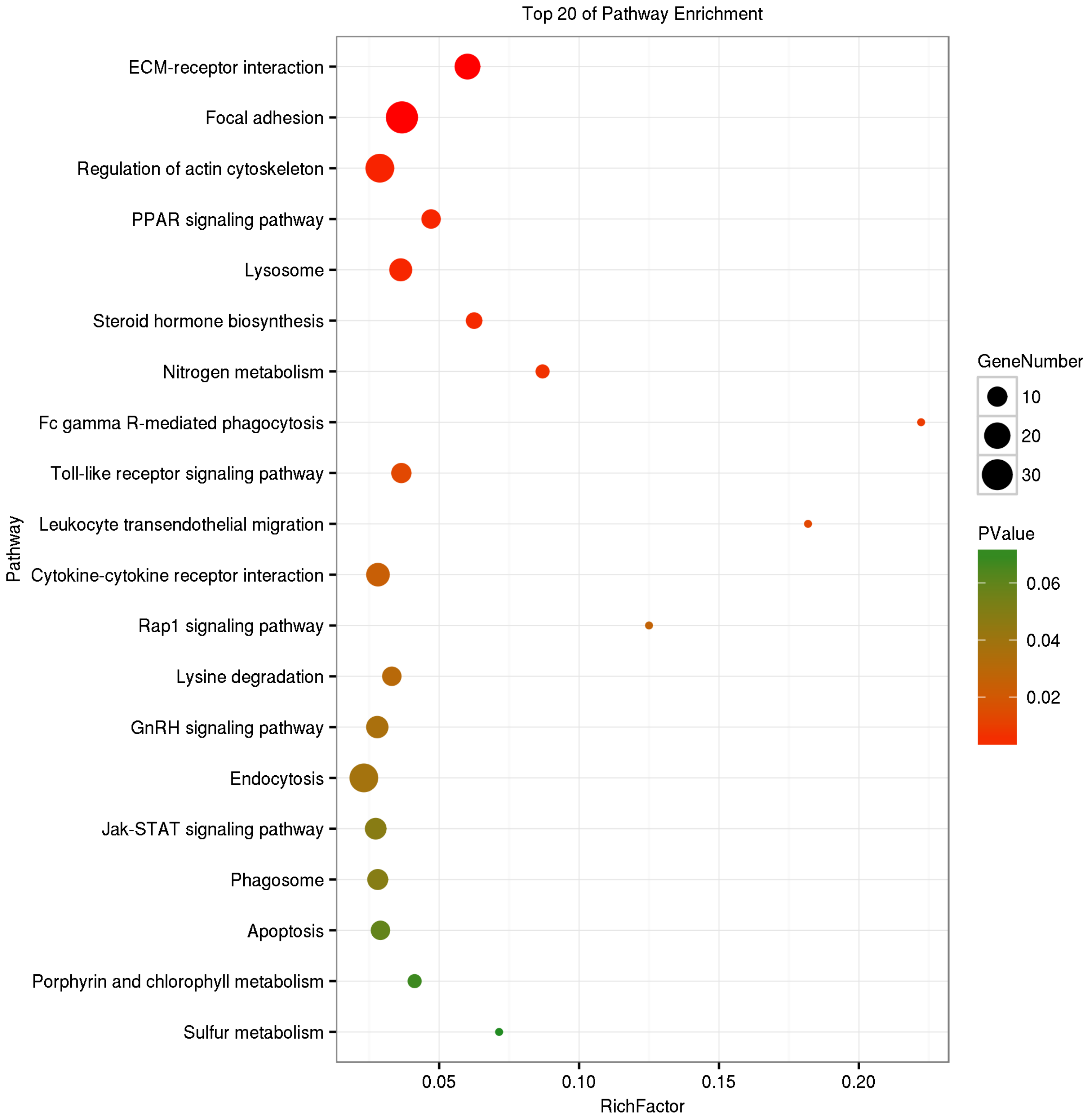

Figure S2. Top 20 significantly enriched pathways associated with genes in ceRNA network.

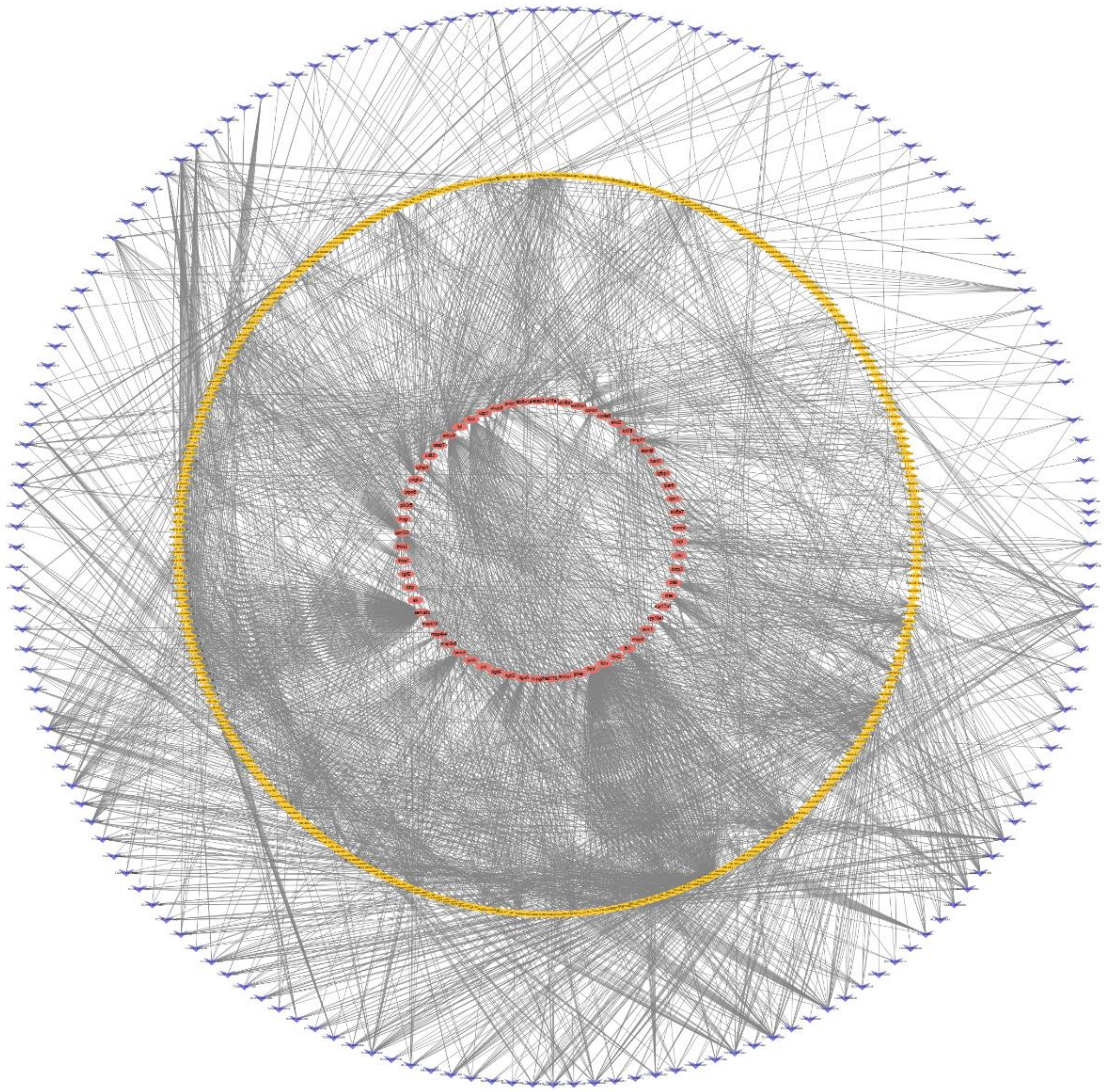

Figure S3. The ceRNA network of genes listed in Table1. Red ellipse represents mRNAs, yellow diamond represents miRNAs and blue “V” represents lncRNAs.

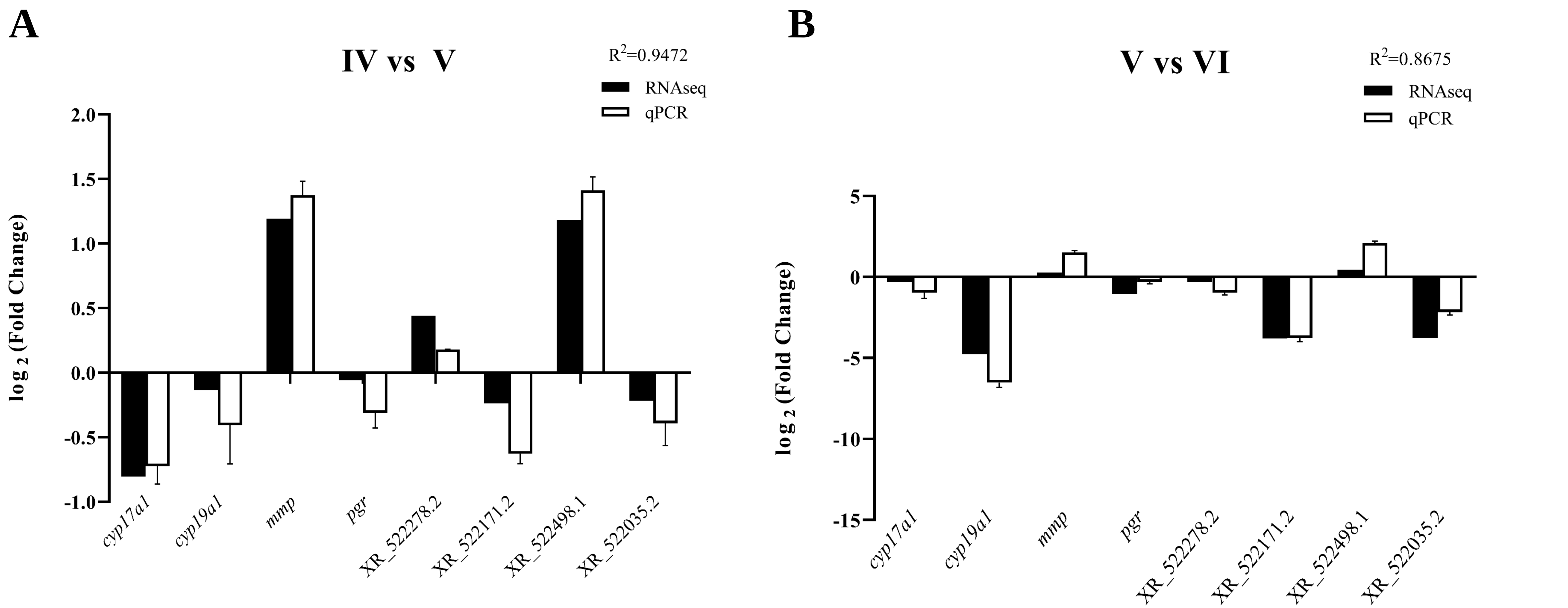

Figure S4. RT-qPCR validation of 4 DE genes (**A**) and 4 DE lncRNAs (**B**) from the tongue sole ovary. The expression levels of the selected genes were normalized to the β-2-m gene.
